# The digital silver lining of the pandemic: The impact on preservice teachers’ technological knowledge and beliefs

**DOI:** 10.1007/s10639-023-11801-w

**Published:** 2023-05-02

**Authors:** Eliana Brianza, Mirjam Schmid, Jo Tondeur, Dominik Petko

**Affiliations:** 1 Institut of Education, University of Zurich, Zurich, Switzerland; 2 University of Queensland, Brisbane, Australia; 3 Vrije Universiteit Brussel, Brussels, Belgium; 4 University of Wollongong, Wollongong, Australia

**Keywords:** TPACK, Technological pedagogical beliefs, Preservice teachers, Teaching experience, COVID-19

## Abstract

COVID-19 drastically disrupted teaching and learning worldwide and across all educational levels. Technology took on a central role in redefining education under these exceptional circumstances and frequently revealed challenges related to both infrastructure and to teachers’ and learners’ technological skills and readiness. This study aimed to investigate whether the experience of emergency remote education significantly impacted preservice teachers’ knowledge and beliefs for their future teaching with technology. We investigated three cohorts of preservice teachers (pre-lockdown, *n* = 179; during lockdown, *n* = 48; post-lockdown, *n* = 228) and explored differences in their self-reported technological pedagogical content knowledge (TPACK) and their technological beliefs. Findings showed positive effects in the post-lockdown cohort, reflected in higher levels of technological knowledge (TK) and technological pedagogical content knowledge (TPCK) compared to the pre-lockdown cohort. In addition, unique positive effects on content knowledge (CK) and pedagogical content knowledge (PCK) were found in the post-lockdown cohort among preservice teachers with prior teaching experiences. No effects of either cohort or experience emerged for preservice teachers’ technological beliefs. These findings indicate that, despite the challenges related to COVID-19 lockdowns, preservice teachers not only appear to have maintained positive beliefs towards technology but may have even been able to draw benefits from the experience of lockdown. These findings and the positive effects associated with teaching experience are discussed with regard to their implication for teacher education.

## Introduction

In the spring of 2020, COVID-19 lockdowns worldwide forced face-to-face teaching and learning activities to shift to remote learning (Meinck et al., 2022). Almost overnight, across school levels and teaching subjects, various forms of delivering instruction emerged, the majority of which saw technological tools take a central stage and often revealed a lack of teachers’ knowledge and skills for using technology for teaching online (Ferri et al., 2020; Marshall et al., 2020). Initial studies report that inservice teachers encountered technical difficulties, challenges in motivating and engaging students (Ewing & Cooper, 2021) and lowered sense of self-efficacy (Ávalos et al., 2022; Cardullo et al., 2021; Pressley & Ha, 2021) frequently leading to negative views towards online teaching during lockdown (DeCoito & Estaiteyeh, 2022). Yet the experiences of the pandemic were not only negative: Teachers reported remote teaching to also be related to heightened flexibility and extension of their pedagogical repetoire to include a range of different resources and ways to support learners, which are not possible in physical classrooms (Cardullo et al., 2021). Similarly, other studies found teachers stating the shift forced them to become more creative and develop new digital skills (DeCoito & Estaiteyeh, 2022; Shamir-Inbal & Blau, 2021). In fact, despite encountering challenges, in their study, DeCoito and Estaiteyeh (2022) found teachers to report intending to integrate more digital elements into their teaching in the coming year. Along these lines, multiple studies conclude that the “new normal” of teaching and learning will be inherently hybrid (e.g., blended learning, flipped classroom), aiming to optimally balance the advantages offered by face-to-face and online learning (Bäcklund et al., 2022; Marek et al., 2021; Shamir-Inbal & Blau, 2021).

Additionally, from the perspective of student achievement, the literature reports contrasting effects of remote learning, ranging from negative (e.g., König & Frey, 2022) to neutral (Tomasik et al., 2021) to positive (e.g., Cavanaugh et al., 2022; Schramm et al., 2021). Particularly with regard to K-12 education, a few studies have found positive outcomes of remote learning on student achievement to be related to 1) students’ familiarity with learning apps and online environments prior to lockdown (König & Frey, 2022) as well as to 2) teachers’ abilities to design high-quality remote instruction (e.g., Clark et al., 2021). Thus, even in returning to “normality”, the road ahead seems to lie in further integration of educational technologies into teaching and learning settings combined with equipping both teachers and students with the knowledge and skills for their use.

The findings described until this point have emphasized the central role of the teacher for effectively adapting to educational disruptions such as the COVID-19 pandemic (e.g., Clark et al., 2021; DeCoito & Estaiteyeh, 2022). Even under “normal” conditions, findings on the integration of technology into educational settings reinforce the centrality of the teachers’ role (e.g., Hixon & Buckenmeyer, 2009; Spiteri & Chang Rundgren, 2020; Tondeur et al., 2017a). Thus, the increasing presence of technologies as both pedagogical tools and practices (i.e., educational technologies) as well as curricular content (i.e., developing students’ knowledge of and skills for using technology; UNESCO, 2021) will naturally reflect on the roles and identities of future teachers. This places a significant focus on understanding how to best prepare current preservice teachers experiencing this transition to the “new normal”. To date, few studies have investigated the impact of the pandemic on how preservice teachers view their professional future. One study among preservice English as a foreign language (EFL) teachers observing online teaching found that preservice teachers reported changes in their identity beliefs for online contexts, as well as heightened appreciation for developing technology integration abilities, and negative views towards online teaching (Gündogdu & Alkayalar, 2021). Romero-Tena et al. (2021) found that students enrolled in an early childhood education course during the pandemic had lower self-reported teaching digital competencies (as described by the authors’ adaptation of the DigCompEdu framework; Redecker, 2017) compared to those having attended the same course the year prior to the pandemic.

Considering the ideal future of education to consist in optimizing the potential of both face-to-face and online teaching and learning, it becomes ever more crucial that post-pandemic teacher training institutions need to prepare prospective teachers for both settings (M. Jin, 2022) and attend to the factors influencing their technology integration, such as their attitudes and beliefs, knowledge and skills, access, and experiences (e.g., Farjon et al., 2019). The literature generally outlines two types of barriers towards technology integration: first order barriers (i.e., challenges related to extrinsic factors and resources such as infrastructure, access, as well as teachers’ knowledge) and second order barriers (i.e., intrinsic obstacles such as teachers’ beliefs; Ertmer, 1999). The present study investigates the relations between the pandemic and relevant teacher-related factors addressing the following research question: How did the pandemic affect preservice teachers’ knowledge (first order barriers) and beliefs (second order barriers) related to teaching with technology? The findings are relevant for teacher training institutions, offering further insight into adequate preparation of future teachers for effectively integrating technology and teaching in the “new normal” educational landscape.

## Overview of the current literature

To better understand the effects of the experience of the COVID-19 pandemic on preservice teachers’ present development and future teaching, we start by reviewing the existing literature describing the challenges and advantages related to these extraordinary circumstances. Subsequently, to situate these findings within the broader field of teacher education, we will focus on two main constructs relevant to preservice teachers’ development and future teaching with technology, namely their technological pedagogical content knowledge (TPACK; Mishra & Koehler, 2006) and the technological beliefs (e.g., Tondeur et al., 2017b; Ertmer & Ottenbreit-Leftwich, 2010).

### Preservice teachers’ experiences of remote education: challenges and advantages

The impact of lockdowns and shifts to remote education was experienced by many preservice teachers on two main activity levels: their *learning* and their *teaching*. This led them to experience remote education from both sides of the coin. From both these perspectives, initial studies find mixed outcomes of preservice teachers reporting both negative as well as positive views towards remote education (e.g., Bäcklund et al., 2022; Elçiçek, 2021; Erumit et al., 2021). With regard to their experiences as remote learners, multiple studies found negative views to be associated with recurring challenges of unreliable or complete lack of infrastructure (Erumit et al., 2021; Mohamad Nasri et al., 2020; Naah, 2020) as well as a lack of motivation (Erumit et al., 2021) and social interaction (Bäcklund et al., 2022). On the positive side, several studies found preservice teachers to report advantages such as saving time and offering greater flexibility as well as being an opportunity for developing new digital skills (Bäcklund et al., 2022) and technological literacy (Elçiçek, 2021). Furthermore, Çevik and Bakioğlu (2022) found that among preservice teachers experiencing remote education in Turkey, their perceived computer self-efficacy was positively related to positive attitudes towards online learning. In contrast, another study reported that preservice teachers who only experienced remote teaching through observing inservice teachers appeared to develop predominantly negative beliefs towards online teaching (Gündogdu & Alkayalar, 2021). Nevertheless, independent of their beliefs, preservice teachers appeared to have a heightened appreciation for the necessity of specialized technological pedagogical skills and knowledge as an integral part of teachers’ identity and their competence repertoire (Gündogdu & Alkayalar, 2021).

With regard to preservice teachers’ teaching experiences, Kadir and Aziz (2021) reviewed the current literature on preservice teachers’ online teaching practicum experiences during emergency remote education and found several factors to influence preservice teachers’ practicum experiences in online settings. Among these, the lack of interaction, knowledge, materials, access, and support from mentor teachers emerged as factors negatively influencing practicum experiences. In contrast, positive online practicum experiences were associated with the development of technological skills and new strategies, support from teacher educators, peer learning, and increased awareness for the socio-cultural contexts of schools and learners (Kadir & Aziz, 2021). In line with these positive findings, Jeh-Awae and Wiriyakarun (2021) found that both personal hands-on online teaching experiences as well as the insight gained from the vicarious experiences of peers enhanced preservice teachers’ self-efficacy.

It is well-established in the literature that positive attitudes and beliefs towards educational technology as well as technological knowledge and skills are key components for successful technology integration (e.g., Li et al., 2019; Petko, 2012, Petko et al., 2017; Prestridge, 2012), particularly since a lack of these factors has been identified as persisting second order barriers in schools in which access and infrastructure are available (i.e., first order barriers; Ertmer et al., 2012). First findings from the experience of the pandemic mirror this pattern, thus reinforcing the call for teacher training institutions to invest greater focus on fostering preservice teachers’ technological knowledge and beliefs in order to adequately prepare them for future practice.

### Teachers’ technological pedagogical content knowledge

**Fig. 1:**
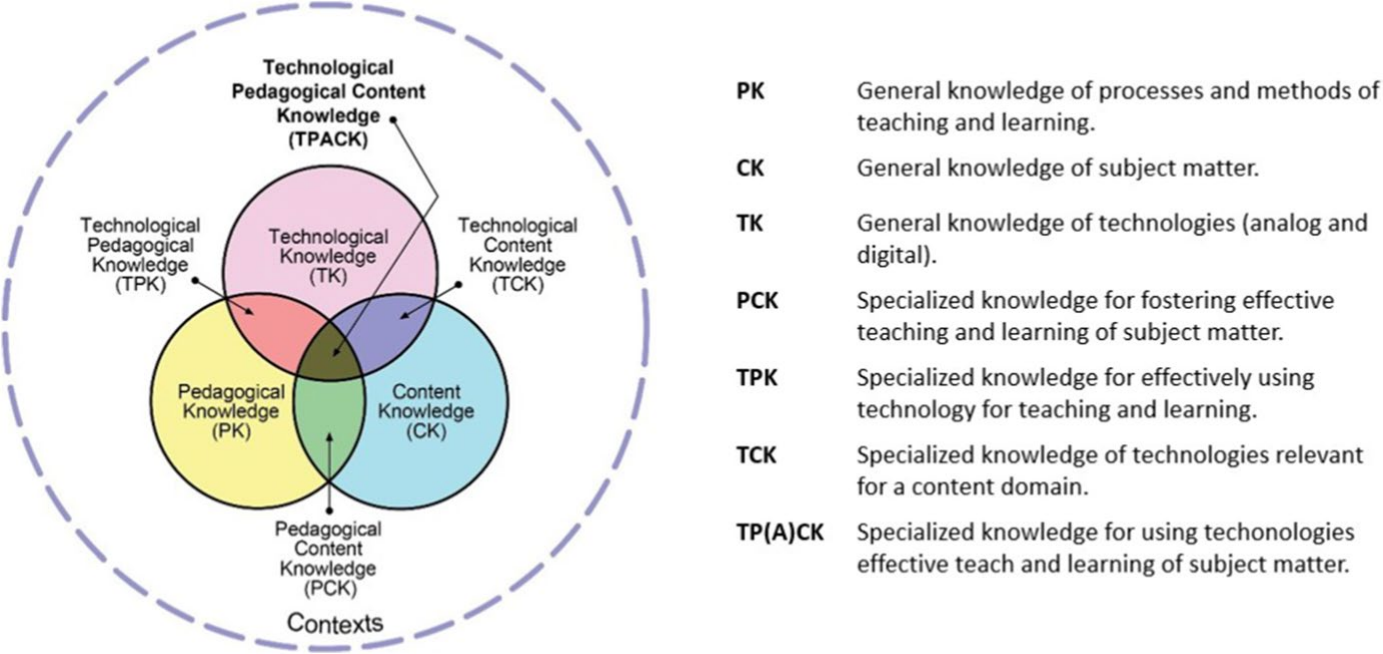
TPACK framework domains and definitions. *Note*. Figure reproduced by permission of the publisher, © 2012 by tpack.org. Definitions of TPACK domains adapted from Mishra and Koehler (2006)

Technology integration in teaching and learning is influenced by an array of factors (e.g., Farjon et al., 2019; Spiteri & Chang Rundgren, 2020), among which a fundamental component consists in teachers’ knowledge for supporting these activities (e.g., Taimalu & Luik, 2019). Although knowledge does not operate in isolation, it is a core component for guiding action (Borko & Putnam, 1996). Knezek and Christensen (2015) found technological pedagogical knowledge to account for 30 percent of the variance of technology integration practices. In response to the new educational opportunities and demands introduced by digital technologies, Mishra and Koehler (2006) proposed an extension to the traditional pedagogical content knowledge framework of teachers’ knowledge (Shulman, 1986, 1987) to include the specific domains of knowledge teachers require for teaching with technology. Their extended technological pedagogical content knowledge (TPACK) framework consists of three core domains (pedagogy, content, and technology) and four hybrid domains arising from their intersections (see Fig. 1; Koehler & Mishra, 2009).

At present, TPACK is a prominent framework in the field of educational technology (Hew et al., 2019) and is adopted for guiding both research (Voogt et al., 2013) and teacher education (Abbitt, 2011; Wang et al., 2018). TPACK has been found to be positively related to teachers’ attitudes towards the pedagogical use of technology (Lee & Tsai, 2010) as well as traceable in lesson plans and practice (Canbazoglu Bilici et al., 2016). Teacher training institutions can effectively foster TPACK’s development (e.g., Y. Jin, 2019; Valtonen et al., 2019), for which they appear to play a crucial role based on findings of the perceived support from teacher training institutions being positively related to preservice teachers’ TPACK, ICT attitudes, and self-efficacy (Petko et al., 2017). In their meta-review of literature reviews, Wang et al. (2018) found three main themes surrounding the development of preservice teachers’ TPACK: 1) the role of effective modeling; 2) the role of experience; and 3) the positive relations of TK with TPACK development. Teacher educators and mentor teachers modeling the effective use of digital technologies in educational settings is a widely acknowledged component in approaches for developing preservice teachers’ TPACK (e.g., TPACK-COPR, Jang & Chen, 2010; TPACK-IDDIRR, C.-J. Lee & Kim, 2014) as well as for preparing them for technology use (e.g., SQD model, Tondeur et al., 2012). In addition, the role of experience is fundamental in learning to teach with educational technology (e.g., Liu, 2012) as well as for developing TPACK (e.g., Jang & Chen, 2010; Tai & Crawford, 2014). Finally, although a person’s general TK does not proportionally translate into TPCK, but rather to an extent it is a unique domain of knowledge (i.e., transformative view; Jin, 2019; Schmid et al., 2020), naturally, the ability to use technology for teaching content implies a degree of TK.

Nevertheless, a recurring issue in TPACK research consists in the gap between individuals’ self-reported knowledge and their performance (e.g., Willermark, 2018). This may partially be due to the fact that, as stated previously, knowledge is not the sole predictor of teachers’ practice. Rather, other factors also play a crucial role and especially considering intricate relations between knowledge and beliefs (e.g., Cheng & Xie, 2018; Tillema, 1995), further insights may be gained by investigating both these factors.

### Teachers’ technology integration beliefs

With regard to teachers’ learning to teach, the literature emphasizes the interplay of both knowledge and beliefs as crucial (e.g., Borko & Putnam, 1996) and this trend is also reflected in studies on preparing teachers for technology integration (e.g., Backfisch et al., 2020; Kim et al., 2013; Taimalu & Luik, 2019). The relations between teachers’ knowledge and their beliefs are not easily disentangled (Ertmer, 2005), as beliefs themselves are the underlying structural systems (i.e., the core concepts that are developed early on and are subsequently self-perpetuating) through which individuals filter information in striving for consensus, thus making them considerably resistant to change (Pajares, 1992). In fact, Calderhead (1996) summarized these two constructs as factual and objective propositions and understandings (i.e., knowledge) that can either be accepted or rejected based on one’s subjective commitments and ideologies (i.e., beliefs). Beliefs and knowledge are thus inextricably tied, yet the self-perpetuating, affective, and instrumental nature of beliefs, makes them predominant drivers of individuals’ behaviors (Pajares, 1992).

With regard to technology integration, in their systematic review Tondeur et al., (2017b) synthesized five main statements characterizing the implications of teachers’ beliefs: 1) The relationship between teachers’ pedagogical beliefs and technology use is bi-directional; 2) teachers’ beliefs can present themselves as barriers to technology integration; 3) specific beliefs are related to types of technology use; 4) professional development plays a crucial role for teachers’ beliefs, as does 5) school context. Additionally, beliefs also appear to have important indirect effects on factors related to technology integration, as shown in the study by Cheng and Xie (2018), which found technological value beliefs to have positive moderating effects on the relationships between teachers’ personal characteristics (i.e., age, gender, and frequency of technology use) and their TPACK. Nevertheless, similarly to the relations between knowledge and performance, several studies among inservice teachers have found that, despite being immersed in technology-rich environments and reporting beliefs valuing technology for promoting student-centered instruction, teachers’ beliefs do not always translate into respective teaching practices (e.g., Ertmer et al., 2012; Palak & Walls, 2009). These studies draw attention to the interplay between beliefs, knowledge, and context for influencing actual performance and emphasize the role of teacher education programs for designing learning experiences for promoting the sophisticated use of technology for teaching and learning.

In fact, experiences are a key component in the formation and consolidation of beliefs (Buehl & Beck, 2015). Among preservice teachers, their experiences as students contribute to shaping their core beliefs on teaching and learning (see Feiman-Nemser, 2001; Pajares, 1992). Nelson and Hawk (2020) found that 1) the experiences of observing technology use by instructors affected preservice teachers’ utility beliefs and intentions to use technology, and that 2) beliefs about the utility (directly) and importance of technology for education (indirectly) predicted their own intentions to use technology. Importantly, their study revealed the quality of these experiences to be crucial, as only in cases in which preservice teachers observed skilled teachers with high TPACK frequently using technology did they find positive effects. In contrast, technology use on part of less skilled teachers or infrequent use on part of highly competent teachers was related to decreased utility and importance beliefs (Nelson & Hawk, 2020).

Considering that within the context of the pandemic preservice teachers were taught remotely and thus experienced and observed use of technology on part of their instructors, these findings would suggest that the quality of the remote education they experienced may have affected their technological beliefs as well as their future intentions to use technology in their own teaching. Thus, as concluded in the previous chapter, the pandemic has not only redesigned the stage of future education, but it also has exposed the future directing actors (i.e., both in- and preservice teachers) to extraordinary experiences which may have shaped fundamental components of their practice. Thus, considering that, despite the challenges of the COVID-19 pandemic, experts have derived lessons learned and directions for the future of education, it is also crucial in parallel to understand the effects this event has had on future teachers, in order to effectively design teacher education tailored to the characteristics and needs of those expected to operate in these advanced educational systems.

### The present study

The present study investigates the effects of the pandemic on preservice teachers’ professional knowledge and beliefs. We explore differences in self-reported TPACK and technological beliefs between cohorts of preservice teachers either before (pre-lockdown cohort), during (lockdown cohort), or after (post-lockdown cohort) the experience of lockdown. In addition, given the importance of experience for both teachers’ knowledge and beliefs, in this study, we include comparisons between preservice teachers based on their prior teaching experience (novices without teaching experience vs. experienced preservice teachers). Based on our research question, investigating the effects of the pandemic on preservice teachers’ TPACK and beliefs based on their level of prior teaching experience, we formulated four hypotheses:

- H1: Compared to the pre-lockdown cohort, the lockdown cohort will have lower technology- and pedagogy-related knowledge domain scores (main effects of cohort).
- H2: Compared to the pre-lockdown cohort, the post-lockdown cohort will have higher technology-related knowledge domain scores (main effects of cohort).
- H3: The effects of H1 and H2 will be stronger for experienced preservice teachers compared to their novice counterparts (effects of the interaction between cohort and experience).
- H4: Experienced preservice teachers’ beliefs will be more stable across cohorts compared to those of their novice counterparts due to their core beliefs having been more consolidated through their prior teaching experiences.

## Methods

### Sample

The sample consisted of 455 preservice upper secondary school teachers enrolled in a compulsory lecture on teaching methodology offered every semester at a Swiss university. As a regular part of this lecture, the same online questionnaire is sent to all course participants one month prior to the end of the semester (i.e., end of May and end of November for the spring and autumn semesters, respectively). Participation in the survey is voluntary and anonymous. Thus, the data used in this study was collected across seven cohorts of preservice teachers: three prior to lockdown (pre-sample), one during lockdown (during-sample), and three after returning to in-person lectures (post-sample). In total we contacted 761 course participants, of which 455 responded (average response rate per semester: 60.2%), resulting in the following final subsamples: pre-sample *n* = 179 (thereof 63 experienced); during-sample *n* = 48 (thereof 17 experienced); and post-sample *n* = 228 (thereof 76 experienced).

### Instruments

#### Teaching experience

In addition to the relevant constructs described below, participants provided demographic information (e.g., gender and age) and their years of previous teaching experience (from 0 = “no previous experience” to 7 = “more than seven years of experience”). Given that the distribution of our sample was highly skewed, with those having no experience accounting for 65.7% of the total sample, we decided to treat experience as a binary variable, namely regarding participants as either “having no teaching experience” (*n* = 299) or “having teaching experience” (i.e., at least one year of teaching experience; *n* = 156).

#### TPACK.xs self-report scale

Participants responded to the TPACK.xs self-report scale (Schmid et al., 2020; see Appendix 1). This scale assesses the seven TPACK domains (four items per domain) on a five-point Likert scale (1 = “strongly disagree”; 5 = “strongly agree”). In our sample, subscales showed good reliabilities across all seven domains (Cronbach’s α: 0.80—0.87).

#### Beliefs about technology

With regard to preservice teachers’ beliefs about teaching with technology, participants responded to two types of beliefs scales: 1) the utility beliefs of ICT for teaching and learning (four items; Petko, 2012; see Appendix 2) and 2) the responsibility of schools for developing students’ digital literacy/awareness (three items; developed by the authors; see Appendix 2). All items were rated on a five-point Likert scale (1 = “strongly disagree”; 5 = “strongly agree”) and both scales showed satisfactory reliabilities (Cronbach’s α: 0.84 and 0.77, respectively).

### Data analysis

To compare our three cohorts and additionally account for experience, we conducted two-way analyses of variance (ANOVAs) to investigate the differences in preservice teachers’ self-reported TPACK domains and beliefs. Prior to the main analysis we checked the required assumptions for conducting ANOVAs of our factors by group (i.e., normality and variance homogeneity). Preliminary analysis revealed significant violations of normality (i.e., skewness and/or kurtosis outside the acceptable range of ± 2; Koh, 2014) for the factors CK and responsibility beliefs, as well as heterogeneity of variance for TPK and TPCK. Thus, for these four factors the non-parametric Scheirer-Ray-Hare test for two-way group comparison (based on the H-statistic) was alternatively adopted (Mangiafico, 2016). Given our unbalanced groups, we adopted the default type II sum of squares estimation (Mangiafico, 2016). Subsequently, in cases of significant ANOVAs (or Scheirer-Ray-Hare tests) post-hoc tests with Tukey correction for multiple comparisons (or Dunn tests with Bonferroni correction for multiple comparisons; Mangiafico, 2016) were conducted to further investigate the source of the group differences. All analyses were conducted in R (version 4.2.0; R Core Team, 2022) using the packages *car* (version 3.1–0; Fox & Weisberg, 2019), *FSA* (version 0.9.3; Ogle et al., 2022) *psych* (version 2.2.5; Revelle, 2022), and *rcompanion* (version 2.4.16; Mangiafico, 2016).

## Results

Investigating our research question for potential effects of the pandemic on preservice teachers’ self-reported TPACK and beliefs, we conducted ANOVAs to assess main effects of cohort and experience as well as the interaction between the two. Regarding our first hypothesis expecting lower scores for pedagogy- and technology- related domains (H1), we found that compared to the TPACK of preservice teachers assessed prior to the pandemic, during lockdown mean ratings were lower on three subscales (i.e., TK, PCK, and TPK; see Table 1). Yet ANOVAs and respective post-hocs tests revealed none of these differences to be significant. Thus, our first hypothesis is rejected.

**Table 1:** TPACK and beliefs variables descriptives by cohort and by experience

		*All cohorts*	Cohort subsamples *M* (*SD*)		
			Pre	During	Post
PK	*All experience levels*	*-*	*3.69 (0.61)*	*3.73 (0.55)*	*3.76 (0.58)*
	novice	*3.59 (0.57)*	3.52 (0.57)	3.68 (0.53)	3.62 (0.57)
	experienced	*4.00 (0.60)*	4.00 (0.71)	3.84 (0.59)	4.03 (0.51)
CK	*All experience levels*	*-*	*4.19 (0.61)*	*4.24 (0.59)*	*4.22 (0.64)*
	novice	*4.14 (0.63)*	4.15 (0.60)	4.34 (0.51)	4.10 (0.67)
	experienced	*4.33 (0.59)*	4.25 (0.62)	4.07 (0.71)	4.46 (0.51)
TK	*All experience levels*	*-*	*3.38 (0.98)*	*3.22 (0.93)*	*3.55 (0.86)*
	novice	*3.38 (0.92)*	3.36 (1.00)	3.10 (0.98)	3.46 (0.84)
	experienced	*3.58 (0.91)*	3.42 (0.96)	3.44 (0.79)	3.74 (0.86)
PCK	*All experience levels*	*-*	*3.87 (0.63)*	*3.80 (0.52)*	*3.91 (0.60)*
	novice	*3.81 (0.58)*	3.78 (0.59)	3.88 (0.54)	3.81 (0.59)
	experienced	*4.02 (0.61)*	4.04 (0.67)	3.66 (0.47)	4.10 (0.56)
TPK	*All experience levels*	*-*	*3.79 (0.71)*	*3.63 (0.82)*	*3.73 (0.60)*
	novice	*3.77 (0.64)*	3.76 (0.67)	3.62 (0.74)	3.81 (0.59)
	experienced	*3.91 (0.70)*	3.86 (0.77)	3.85 (0.78)	3.97 (0.63)
TCK	*All experience levels*	*-*	*3.26 (1.00)*	*3.29 (0.92)*	*3.35 (0.88)*
	novice	*3.30 (0.91)*	3.31 (0.94)	3.25 (0.88)	3.31 (0.89)
	experienced	*3.32 (0.98)*	3.17 (1.10)	3.37 (1.02)	3.44 (0.86)
TPCK	*All experience levels*	*-*	*3.55 (0.77)*	*3.63 (0.82)*	*3.73 (0.60)*
	novice	*3.60 (0.68)*	3.50 (0.73)	3.56 (0.84)	3.68 (0.59)
	experienced	*3.74 (0.73)*	3.63 (0.83)	3.74 (0.79)	3.84 (0.62)
Beliefs_utility	*All experience levels*	*-*	*3.69 (0.80)*	*3.72 (0.66)*	*3.71 (0.71)*
	novice	*3.71 (0.68)*	3.68 (0.74)	3.71 (0.64)	3.75 (0.64)
	experienced	*3.71 (0.85)*	3.71 (0.90)	3.72 (0.72)	3.70 (0.84)
Beliefs_resp	*All experience levels*	*-*	*4.45 (0.66)*	*4.38 (0.60)*	*4.36 (0.70)*
	novice	*4.43 (0.64)*	4.48 (0.64)	4.37 (0.60)	4.40 (0.65)
	experienced	*4.34 (0.73)*	4.40 (0.70)	4.41 (0.63)	4.27 (0.77)

Subsamples size: pre-novice *n* = 116; pre-experienced *n* = 63; during-novice *n* = 31; during-experienced *n* = 17; post-novice *n* = 152; post-experienced *n* = 76

We did find significant cohort main effects for TK (*F*(1) = 3.62, *p* = 0.028) as well as for TPCK (*H*(1) = 6.67, *p* = 0.036). Post-hoc tests revealed these to arise from significantly higher post- compared to both pre- and during-lockdown cohorts for TK and between pre- with post-lockdown cohorts for TPCK (see Table 2). The increase in these two domains is consistent with and partially confirms our second hypothesis (H2), for which we expected higher scores in technology-related domains among our post-lockdown cohort compared to the other two groups. In addition, we found main effects of experience. Experienced preservice teachers scored higher than their novice counterparts on all seven domains (see Table 1), among which their scores on five domains were significantly higher: PK (*F*(1) = 23.97, *p* < 0.001), CK (*H*(1) = 11.15, *p* = 0.001), TK (*F*(1) = 4.24, *p* = 0.040), TPK (*H*(1) = 5.75, *p* = 0.017), and TPCK (*H*(1) = 4.52,* p* = 0.033).

**Table 2:** Summary of significant effects of cohort, experience, and their interaction

Predictors	Dependent variable	Significant post hoc group differences				Correction for multiple comparisons	
Cohort	TK	Post	>	Pre	*p* = 0.032	*p*_Tukey_	= 0.082
			>	During	*p* = 0.031	*p*_Tukey_	= 0.079
	TPCK	**Post**	** > **	**Pre**	***p* = 0.011**	***p***_**Bonferroni**_	** = 0.033**
Experience	PK	**Exp**	** > **	**Inexp**	***p* < 0.001**	***p***_**Tukey**_	** < 0.001**
	CK	**Exp**	** > **	**Inexp**	***p* < 0.001**	***p***_**Bonferroni**_	** < 0.001**
	TK	**Exp**	** > **	**Inexp**	***p* = 0.040**	***p***_**Tukey**_	** = 0.040**
	TPK	**Exp**	** > **	**Inexp**	***p* = 0.017**	***p***_**Bonferroni**_	** = 0.017**
	TPCK	**Exp**	** > **	**Inexp**	***p* = 0.038**	***p***_**Bonferroni**_	** = 0.038**
Cohort*Experience	CK	**Post-exp**	** > **	**Pre-inexp**	***p* < 0.001**	***p***_**Bonferroni**_	** = 0.004**
			** > **	**Post-inexp**	***p* < 0.001**	***p***_**Bonferroni**_	** < 0.001**
			>	Pre-exp	*p* = 0.029	*p*_Bonferroni_	= 0.432
			>	During-exp	*p* = 0.002	*p*_Bonferroni_	= 0.227
	PCK	Pre-exp	>	Pre-inexp	*p* = 0.007	*p*_Tukey_	= 0.073
			>	Post-inexp	*p* = 0.011	*p*_Tukey_	= 0.114
			>	During-exp	*p* = 0.021	*p*_Tukey_	= 0.189
		**Post-exp**	** > **	**Pre-inexp**	***p* < 0.001**	***p***_**Tukey**_	** = 0.005**
			** > **	**Post-inexp**	***p* < 0.001**	***p***_**Tukey**_	** = 0.009**
			>	During-exp	*p* = 0.006	*p*_Tukey_	= 0.070

Differences remaining significant after Tukey or Bonferroni correction for multiple comparisons are presented in boldface

Subsequently, investigating the interaction between cohort and experience, findings showed experienced preservice teachers to have the highest scores across TPACK domains, among which we found significant effects for the domains of CK and PCK. For CK, post-hoc tests showed that experienced preservice teachers reported significantly higher scores after lockdown compared to novice preservice teachers pre- and post-lockdown, as well as compared to experienced preservice teachers’ scores pre- and during-lockdown (see Table 2). With regard to PCK, findings showed that although novice preservice teachers tended to rate themselves higher during lockdown compared to their ratings prior to and after lockdown (see Table 1), overall, their scores were not significantly affected. In contrast, lockdown appeared to affect experienced preservice teachers, who showed significantly lower ratings during lockdown compared to both pre- and post-lockdown cohorts (see Table 2). Thus, our third hypothesis expecting stronger effects of lower pedagogy- and technology-related domains during lockdown for experienced compared to novice preservice teachers (H3), is partially confirmed for the domain of PCK. A final observation regarding TPACK: The only domain for which no effects of any predictors were found, was for that of TCK.

With regard to preservice teachers’ technological beliefs, no effects of cohort or experience emerged for either utility or responsibility beliefs (see Table 1). Interestingly, investigating the single items for group differences we found that only one item on the utility beliefs scale (i.e., “By using digital technologies, I can improve the quality of my teaching”; see Appendix 2, item bp1) showed a significant main effect of cohort (*F*(2) = 3.910, *p* = 0.021), with post-hoc tests showing this difference to arise from the comparison of pre- and post-lockdown scores (*p* = 0.006, *p*_Tukey_ = 0.015).

## Discussion

The COVID-19 pandemic caused undeniable disruptions to education worldwide. Yet despite the struggles and challenges, the experience also resulted in some positive consequences, increasing insight into the potential of technologies for education across contexts (e.g., DeCoito & Estaiteyeh, 2022; Elçiçek, 2021). In this study we aimed to shed more light on how the pandemic affected preservice teachers’ evaluations of their professional knowledge for teaching in the digital era as well as their beliefs on the utility of technology for education and the responsibility of education systems for developing learners technological competences. Overall, four main findings emerged from our study. First, we found that, compared to preservice teachers prior to the pandemic, after the experience of lockdown, both novice and experienced preservice teachers appear more confident in their general technological knowledge (i.e., TK) as well as in their subject-specific knowledge for teaching with technology (i.e., TPCK). Second, consistent with the literature (e.g., Tai & Crawford, 2014; Wang et al., 2018), we replicated findings of the positive effects of experience on preservice teachers’ TPACK, showing that, except for the domains PCK and TCK, those with experience had significantly higher TPACK scores compared to inexperienced novices. Third, we found initial evidence of experience-related advantages for CK and PCK, as post-lockdown experienced preservice teachers’ ratings for these domains were significantly higher than those of pre- and post-lockdown novices. Finally, the pandemic does not appear to have impacted preservice teachers’ beliefs on technology in educational contexts.

Our first finding of a significant cohort effect on TPACK relates to our first two hypotheses. Firstly, compared to prior to the pandemic, we expected preservice teachers during lockdown to experience challenges to their technology- and pedagogy-related domains, resulting in lower scores for these domains (H1). This hypothesis was rejected, as we found no significant decreases in TPACK domains between pre- and during-lockdown scores. Nevertheless, it is interesting to note, that although these differences did not quite reach significance, experienced preservice teachers revealed patterns in line with this first hypothesis, as the during-lockdown cohort showed a drop in PK, CK, and PCK. In contrast, inexperienced novices showed inverse tendencies across cohorts, revealing slight increases in these domains among the during- compared to pre- and post-lockdown cohorts. Drawing on research investigating inservice and preservice teachers’ teaching experiences during lockdown, several studies found that in addition to technological challenges, teachers mentioned pedagogy-related challenges such as engaging and motivating students (DeCoito & Estaiteyeh, 2022; M. Jin, 2022; Marshall et al., 2020), integrating collaborative learning approaches (Mohamad Nasri et al., 2020), as well as assessing and holding students accountable for their work (DeCoito & Estaiteyeh, 2022; Marshall et al., 2020; Mohamad Nasri et al., 2020). These findings could suggest that preservice teachers with previous teaching experience may be more susceptible to the interdependent complexity of educational settings and perceive shifts that appear to be predominantly technology-related to give rise to pedagogical challenges. In contrast, given their lack of experience in educational settings from a teachers’ perspective, novices may yet lack this sophisticated understanding of the strong interrelations between contextual factors and practice (see Brianza et al., 2022; Mishra & Warr, 2021).

Our second hypothesis expected preservice teachers post-lockdown to have gained new technological knowledge and thus report higher scores on their technology-related domains (H2). This hypothesis could be partially confirmed, as we found fundamental technology-related domains (TK and TPCK) to be higher in the post-lockdown cohort compared to in the pre-lockdown. Interestingly, no effects of lockdown were found for TCK and TPK. This finding is in line with the conception of TPACK as a transformative construct (e.g., Angeli & Valanides, 2005; Mishra & Koehler, 2006; Schmid et al.,^ 2020^) consisting of unique knowledge domains, with its hybrid domains being more than the summation of the core factors. In relation to this transformative view, the fact that we did not find effects of the experience of lockdown on TCK and TPK may be related on one hand, to the specificity of this experience—during which teachers mostly applied technology and pedagogy to their teaching subject (i.e., TPCK)—and, on the other hand, to the limited duration of this experience—proving too short to accumulate a wider range of experiences for developing more generic understandings of technology’s value for pedagogy (i.e., TPK) and content (i.e., TCK).

As a final point regarding the effects of the experience of lockdown, although these findings are only cross-sectional rather than longitudinal and are thus limited in their interpretation (see Section 6 for further discussion), they present initial evidence suggesting that preservice teachers may have drawn some benefits for their technology-related knowledge from the experience of remote learning. Several further studies report similar findings of the experience of lockdown to have been an opportunity for both inservice (e.g., DeCoito & Estaiteyeh, 2022) as well as preservice teachers (e.g., Bäcklund et al., 2022; Elçiçek, 2021) to extend their knowledge of technological tools and resources, as well as being forced to develop new designs and approaches in their teaching. Taken together, evidence presents the experience of lockdown as an event giving rise to a period effect (i.e., experience similarly affecting all groups within a population; see Altman, 2014) and thus of relevance for comparative research as well as for the professional development of preservice and inservice teachers having experienced lockdowns.

With regard to our third hypothesis (i.e., expecting the effects of lockdown to be stronger among experienced preservice teachers compared to novices), we only found effects of the interaction between cohort and experience for the domains of CK and PCK, suggesting that experienced preservice teachers drew benefits from the experience of lockdown that novice preservice teachers did not grasp (partially confirming H3). In contrast and partially rejecting our hypothesis, no effects emerged for the technology-related domains. This indicates that the higher scores on TK and TPCK among preservice teachers having experienced lockdown were unrelated to prior teaching experience. Considering that the literature generally describes teaching experience to benefit preservice teachers’ knowledge development through supporting them in making connections between theory and practice (e.g., Darling-Hammond, 2006; Korthagen & Kessels, 1999), our findings align with this assumption with regard to preservice teachers’ knowledge of their subject matter (i.e., CK) and how to teach it (i.e., PCK). From this perspective, the unusual teaching and learning circumstances of lockdown might have challenged experienced preservice teachers to critically consider their CK and PCK from different perspectives and draw connections with their previously developed knowledge. Novices, in contrast, did make connections between the experience of remote education and more distal knowledge domains, but rather only matched their experienced counterparts in grasping the “surface” aspects related to technology.

Finally, addressing our fourth hypothesis on the stability of preservice teachers’ technological pedagogical beliefs across cohorts, our findings confirmed this expectation showing no changes across cohorts in self-rated beliefs. Contrary to our expectations, there was no difference between experience groups. These findings are particularly interesting for several main reasons: Firstly, from a methodological perspective, finding different patterns of effects for knowledge compared to belief measures reflects the nature of these constructs reported in the literature and thus supports these constructs as distinct as well as the validity of our measures. Secondly, it suggests that, although lockdowns drastically disrupted education on a global level, despite the significant challenges of this period our future teachers’ beliefs towards educational technology and their high regard for technology’s role in education remained intact, reflecting the nature of beliefs as relatively stable and not easily changed personal constructs (see Pajares, 1992). This is important given that research presents technological pedagogical beliefs to be related to aspects of technology use in educational settings (e.g., Bahcivan et al., 2019; Kim et al., 2013).

Overall, considering that in educational settings technology will only become increasingly more present (Dishon, 2021), future teachers need to develop the respective knowledge and beliefs for supporting effective integration of technologies into their teaching and learning activities (Starkey, 2020). Given that teacher training plays a crucial role for developing teachers’ TPACK (e.g., Wang et al., 2018), shaping their beliefs (e.g., Nelson et al., 2020), and preparing them to teach with technology (Tondeur et al., 2012), it is positioned at the frontline of advancing teaching and learning within the “new normal”. Furthermore, the need for specialized and continuous training to keep up with the pace of technological developments or possible future disruptions is echoed even among inservice teachers by findings such as those of Scherer et al. (2023), who found a curvilinear relationship between prior online teaching experience and teachers’ readiness to teach online during the COVID-19 pandemic. Thus, it is no longer sufficient for teacher education to prepare preservice teachers for face-to-face instruction, but rather it also needs to incorporate sufficient experiences with current technologies and their relevant discussions in educational settings.

## Limitations and future research

The findings of this study need to be interpreted under the lens of several main limitations. First, this study did not use longitudinal data, thus we cannot make direct inferences on the effects of lockdown experiences on knowledge and beliefs, but rather these need to be viewed as cross-sectional comparisons. Second, for both constructs we relied on self-reported data, which in addition to the gaps between self-reported both TPACK and beliefs in relation to teaching practice noted above, are subject to an array of biases (Paulhus & Vazire, 2007). This point is particularly important to consider with regard to knowledge constructs (Park et al., 1988), as an individual’s ability to report on one’s own knowledge is unavoidably affected by the very level of one’s knowledge (see Dunning-Kruger effect, Kruger & Dunning, 1999). Third, particularly with respect to beliefs, the literature emphasizes the importance of the quality of experiences for shaping one’s beliefs (e.g., Nelson & Hawk, 2020). In this study we were not able to investigate aspects related to the quality of our preservice teachers’ lockdown experience and thus, we cannot exclude that accounting for differences in the quality of experiences may have revealed other effects on beliefs. Finally, among experienced preservice teachers it was again not possible to control for the type or quality of their prior teaching experiences or for whether they were actively teaching during lockdowns. Future research is required to address these four points and more carefully investigate the mechanisms that support preservice teachers in assimilating professional knowledge through observing teaching and experiencing learning in exceptional situations.

## Conclusions

This study aimed to contribute to the literature investigating the consequences of the extraordinary experience of the COVID-19 pandemic on educational systems and specifically on those who will be leading our future classrooms, namely preservice teachers. Despite the disruptions and challenges related to the COVID-19 pandemic, our study adheres to those revealing a silver lining of these experiences, outlining potential benefits for moving forward in the “new normal”. Our findings indicate that the drastic conditions of lockdown did not negatively affect preservice teachers’ confidence in their knowledge or their technology-related educational beliefs. Rather preservice teachers appear to have even learned from this experience and maintained their core beliefs. In addition, the current study provides evidence of the value of prior experiences for supporting learning under exceptional circumstances, as we found experienced preservice teachers to draw benefits for their TPACK, whereas inexperienced novices appear to have “missed” this opportunity. Thus, particularly with regard to training preservice teachers without prior classroom experience, teacher education institutions should place considerable effort into incorporating methods emphasizing authentic teaching and learning experiences and supporting preservice teachers in developing abilities for “seeing” the connections between theory and practice even under exceptional circumstances. Finally, seizing the opportunity to maximize what we have learned from this (hopefully) exceptional experience of the pandemic, moving into the “new normal” must include preparing teachers for a blended educational landscape.

## Data Availability

The datasets generated during and/or analysed during the current study are available from the corresponding author on reasonable request.

## Appendix 1

Table 3

**Table 3:** TPACK.xs scale descriptives and reliability

Item		(Sub)sample *M(SD)*			
		*full*	pre	during	post
pk1	I can adapt my teaching based upon what students currently understand or do not understand	*3.83 (0.73)*	3.78 (0.76)	3.92 (0.68)	3.85 (0.71)
pk2	I can adapt my teaching style to different learners	*3.68 (0.77)*	3.61 (0.84)	3.77 (0.59)	3.72 (0.74)
pk3	I can use a wide range of teaching approaches in a classroom setting	*3.62 (0.79)*	3.56 (0.87)	3.54 (0.82)	3.68 (0.72)
pk4	I can assess student learning in multiple ways	*3.79 (0.82)*	3.80 (0.81)	3.71 (0.85)	3.80 (0.82)
PK scale Cronbach’s α:		*0.80*			
ck1	I have sufficient knowledge about my teaching subject	*4.25 (0.80)*	4.24 (0.82)	4.23 (0.69)	4.27 (0.81)
ck2	I can use a subject-specific way of thinking in my teaching subject	*4.37 (0.73)*	4.36 (0.74)	4.40 (0.61)	4.38 (0.76)
ck3	I know the basic theories and concepts of my teaching subject	*4.36 (0.68)*	4.34 (0.66)	4.35 (0.67)	4.37 (0.69)
ck4	I know the history and development of important theories in my teaching subject	*3.85 (0.92)*	3.80 (0.94)	4.00 (0.90)	3.86 (0.90)
CK scale Cronbach’s α:		*0.80*			
tk1	I keep up with important new technologies	*3.52 (0.92)*	3.44 (1.14)	3.29 (1.03)	3.62 (1.01)
tk2	I frequently play around with technology	*3.04 (1.20)*	2.96 (1.23)	2.81 (1.20)	3.16 (1.17)
tk3	I know about a lot of different technologies	*3.41 (1.04)*	3.37 (1.07)	3.13 (1.04)	3.50 (1.01)
tk4	I have the technical skills I need to use technology	*3.83 (0.94)*	3.76 (1.04)	3.65 (0.96)	3.92 (0.85)
TK scale Cronbach’s α:		*0.89*			
pck1	I know how to select effective teaching approaches to guide student thinking and learning in my teaching subject	*3.79 (0.73)*	3.82 (0.76)	3.71 (0.65)	3.79 (0.72)
pck2	I know how to develop appropriate tasks to promote students’ complex thinking of my teaching subject	*3.87 (0.76)*	3.85 (0.79)	3.79 (0.71)	3.91 (0.74)
pck3	I know how to develop exercises with which students can consolidate their knowledge of my teaching subject	*3.98 (0.75)*	3.99 (0.75)	3.92 (0.65)	4.00 (0.77)
pck4	I know how to evaluate students’ performance in my teaching subject	*3.87 (0.80)*	3.83 (0.82)	3.79 (0.74)	3.93 (0.79)
PCK scale Cronbach’s α:		*0.80*			
tpk1	I can choose technologies that enhance the teaching approaches for a lesson	*3.78 (0.81)*	3.74 (0.89)	3.60 (0.89)	3.84 (0.72)
tpk2	I can choose technologies that enhance students’ learning for a lesson	*3.63 (0.83)*	3.58 (0.93)	3.58 (0.87)	3.68 (0.73)
tpk3	I can adapt the use of the technologies that I am learning about to different teaching activities	*4.13 (0.83)*	4.18 (0.78)	3.98 (0.93)	4.11 (0.84)
tpk4	I am thinking critically about how to use technology in my classroom	*3.75 (0.87)*	3.67 (0.95)	3.65 (0.93)	3.82 (0.78)
TPK scale Cronbach’s α:		*0.81*			
tck1	I know how technological developments have changed the field of my subject	*3.52 (1.08)*	3.51 (1.08)	3.65 (1.00)	3.50 (1.09)
tck2	I can explain which technologies have been used in research in my field	*3.45 (1.12)*	3.44 (1.18)	3.33 (1.10)	3.48 (1.08)
tck3	I know which new technologies are currently being developed in the field of my subject	*2.93 (1.14)*	2.89 (1.20)	2.85 (1.24)	2.97 (1.07)
tck4	I know how to use technologies to participate in scientific discourse in my field	*3.34 (1.04)*	3.20 (1.12)	3.33 (1.15)	3.46 (0.94)
TCK scale Cronbach’s α:		*0.87*			
tpck1	I can use strategies that combine content, technologies, and teaching approaches that I learned about in my coursework in my classroom	*3.70 (0.76)*	3.67 (0.84)	3.71 (0.85)	3.71 (0.67)
tpck2	I can choose technologies that enhance the content for a lesson	*3.51 (0.90)*	3.40 (0.96)	3.40 (0.94)	3.63 (0.83)
tpck3	I can select technologies to use in my classroom that enhance what I teach, how I teach, and what students learn	*3.73 (0.86)*	3.59 (0.94)	3.79 (0.92)	3.82 (0.76)
tpck4	I can teach lessons that appropriately combine my teaching subject, technologies, and teaching approaches	*3.66 (0.82)*	3.53 (0.86)	3.60 (0.96)	3.77 (0.74)
TPCK scale Cronbach’s α:		*0.86*			

## Appendix 2

Table 4

**Table 4:** Beliefs scales descriptives and reliability

Item		(Sub)sample *M(SD)*			
		*full*	pre	during	post
*Utility beliefs of ICT for teaching and learning* ^*1*^					
bp1	By using digital technologies, I can improve the quality of my teaching	*4.00 (0.85)*	3.85 (0.90)	3.96 (0.68)	4.12 (0.82)
bp2	Using computers in class can raise student performance	*3.62 (0.91)*	3.61 (0.94)	3.65 (0.86)	3.62 (0.90)
bp3	By using digital technologies, students’ learning and working strategies can be improved	*3.66 (0.89)*	3.72 (0.92)	3.69 (0.83)	3.61 (0.88)
bp4	By using digital technologies, students work more efficiently	*3.55 (0.94)*	3.60 (0.94)	3.58 (0.85)	3.50 (0.96)
Cronbach’s α:		*0.84*			
*Responsibility beliefs for developing digital literacy* ^*2*^					
br1	It is important that all students learn how to use digital media responsibly in the classroom	*4.61 (0.69)*	4.61 (0.67)	4.60 (0.70)	4.60 (0.70)
br2	Schools are tasked with developing students’ critical attitudes towards the possible side effects of new technologies	*4.40 (0.80)*	4.47 (0.79)	4.38 (0.76)	4.35 (0.81)
br3	As a teacher I must make students aware of the role digital technologies play in our society	*4.19 (0.92)*	4.27 (0.92)	4.17 (0.88)	4.13 (0.93)
Cronbach’s α:		*0.77*			

^1^ Adapted from Petko (2012). ^2^ Developed by the authors
